# Strengthening awareness and response to HTLV‐1 infection in Africa: A neglected threat to blood safety and public health

**DOI:** 10.1002/hem3.70234

**Published:** 2025-10-07

**Authors:** Jean‐Claude Twizere, Carolina Rosadas, Maureen Kidiga, Fatumata Djalo, Edward L. Murphy, Augustin Mouinga‐Ondeme, Marion Vermeulen, Louine Morrell, Saliou Diop, Sibusiso Maseko, Peregrine Sebulime, Andrews Akwasi Agbleke, Boineelo Fundisi, Espérance Umumararungu, Patricia Watber, Carol Hlela, Egídio Nhavene, Thato Chidarikire, Olivier Hermine, Antoine Gessain

**Affiliations:** ^1^ Viral Interactome Lab, Computational and Molecular Biology Unit, GIGA Institute University of Liege Liege Belgium; ^2^ Imperial College London London UK; ^3^ Kyoto University Kyoto Japan; ^4^ University of California San Francisco California USA; ^5^ Centre Interdisciplinaire de Recherches Médicales de Franceville Unité des Infections Rétrovirales et Pathologies Associées Franceville Gabon; ^6^ The South African National Blood Service (SANBS) Roodeport Gauteng South Africa; ^7^ Communicable Disease Control Unit Seychelles Hospital Victoria Seychelles; ^8^ University Cheikh Anta Diop, and National Blood Transfusion center Dakar Senegal; ^9^ Makerere University Kampala Uganda; ^10^ Sena Institute of Technology Ketu Ghana; ^11^ HIV Testing Services Program, Department of Public Health Ministry of Health Gaborone Botswana; ^12^ Red Cross Children's Hospital University of Cape Town Cape Town South Africa; ^13^ Instituto Nacional de Saude Maputo Mozambique; ^14^ National HIV Prevention World Health Organization Pretoria South Africa; ^15^ Department of Haematology and INSERM U1163, Imagine Institute Hôpital Necker and Paris cité University, Inidex GRex Paris France; ^16^ Unité d'Epidémiologie et Physiopathologie des Virus Oncogènes, Institut Pasteur CNRS UMR Paris France

Human T‐cell lymphotropic virus type 1 (HTLV‐1) is a deltaretrovirus endemic in several regions worldwide, with Africa considered the largest reservoir of infection.^1^, ^2^ Despite its discovery over 45 years ago,^3^, ^4^ HTLV‐1 remains one of the most neglected infectious agents in global public health. It is estimated that at least 5 to 10 million individuals are infected worldwide, with a disproportionately high prevalence concentrated in sub‐Saharan Africa, particularly in western, central, and austral regions.^1^, ^5^

HTLV‐1 is a life‐long infection, transmitted primarily via mother‐to‐child (especially through prolonged breastfeeding), sexual contact (especially from men to women), and blood transfusion or organ transplantation. The virus is etiologically linked to a spectrum of diseases including adult T‐cell leukemia/lymphoma (ATLL), a highly aggressive hematological malignancy with poor prognosis,^6^ and HTLV‐1‐associated myelopathy/tropical spastic paraparesis (HAM/TSP), a chronic, disabling neurological disorder.^7^ There is also strong evidence that HTLV‐1 infection is associated with increased age‐adjusted all‐cause mortality.^8^ Despite these severe outcomes, most infected individuals remain asymptomatic throughout life, or die of fulminant hematological malignancies before the diagnosis is made, complicating public health surveillance and awareness efforts.

Unfortunately, routine testing for HTLV‐1 remains absent or extremely limited in most African countries, including testing prior to blood transfusions, a key route of transmission.^9^ This perspective article synthesizes the first symposium on HTLV‐1 and blood transfusion in Africa, including current knowledge on HTLV‐1 epidemiology, clinical impact, and research advances on the continent. It further highlights critical gaps in diagnostic and preventive policies, culminating in a summary of key policy recommendations to strengthen regional responses.

## HTLV‐1 EPIDEMIOLOGY IN AFRICA: A PATCHWORK OF DATA AND HIGH ENDEMICITY

**Figure 1:**
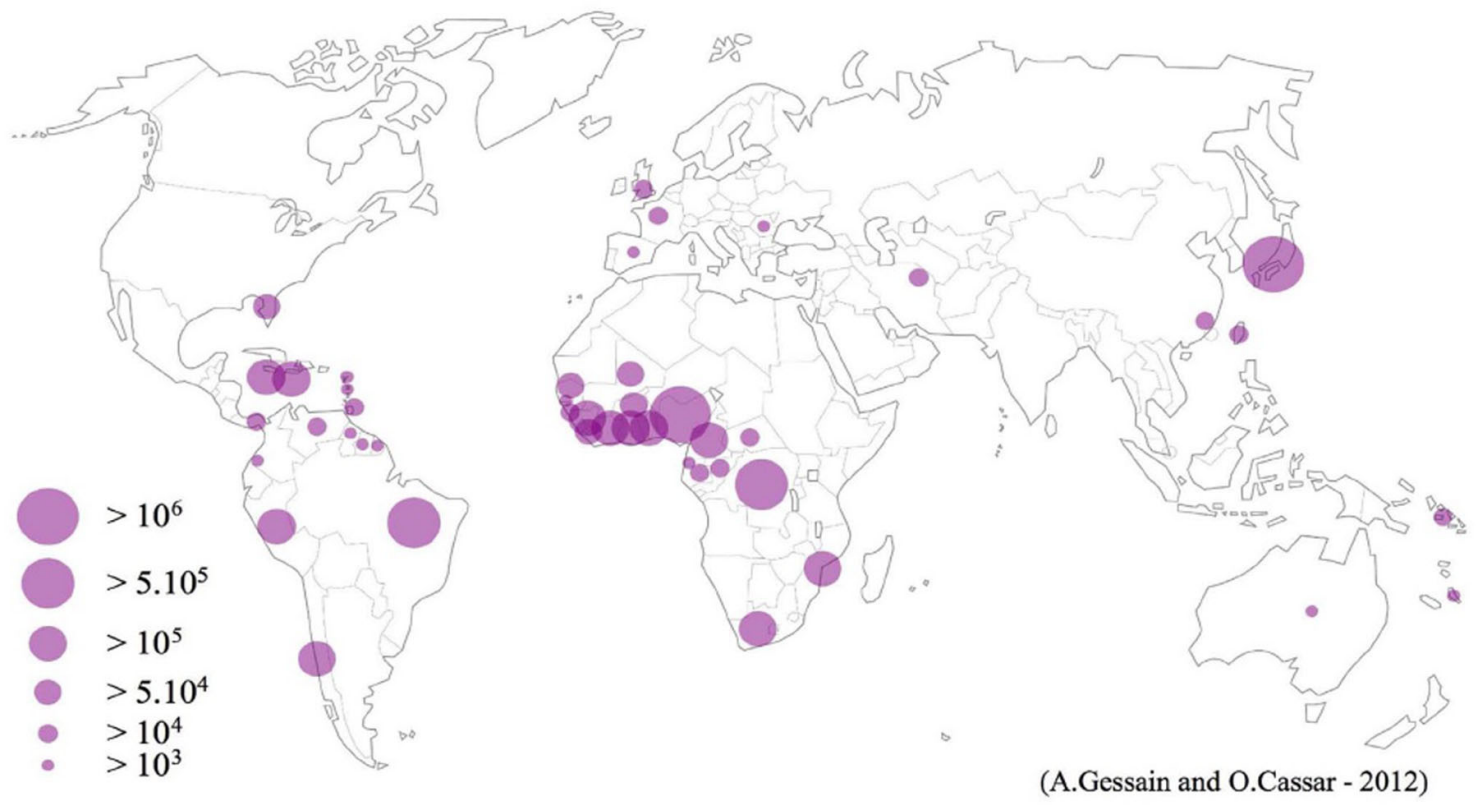
**World distribution of HTLV‐1 infection**.

Africa harbors a complex and heterogeneous HTLV‐1 epidemiological landscape. The virus's distribution in Africa is highly uneven: high‐prevalence clusters exist in western, central, and southern Africa, while northern and eastern regions show relatively lower infection rates. The highest adult prevalence in some rural areas of Gabon and the Democratic Republic of Congo (DRC) reaches between 10% and 25%, particularly among older women,^1^, ^5^ and several cases of HTLV‐1‐associated diseases were already reported in Africa^1^, ^2^, ^5^ (Figure 1). However, reliable prevalence data remain sparse across many African countries due to limited large‐scale, representative studies and frequent reliance on single‐step serological assays without confirmatory testing. This results in frequent overestimation or underestimation of true infection rates, hampering accurate mapping and public health prioritization. The viral genetic diversity in Africa is remarkable, with six distinct HTLV‐1 genotypes (a, b, d, e, f, g) identified. Genotype b predominates in central Africa, while the cosmopolitan genotype a dominates West and South Africa.^5^ Understanding this genetic heterogeneity is crucial for epidemiological tracking and may provide insights into differential disease risks.

## CLINICAL SPECTRUM AND CHALLENGES IN DIAGNOSIS

**Figure 2:**
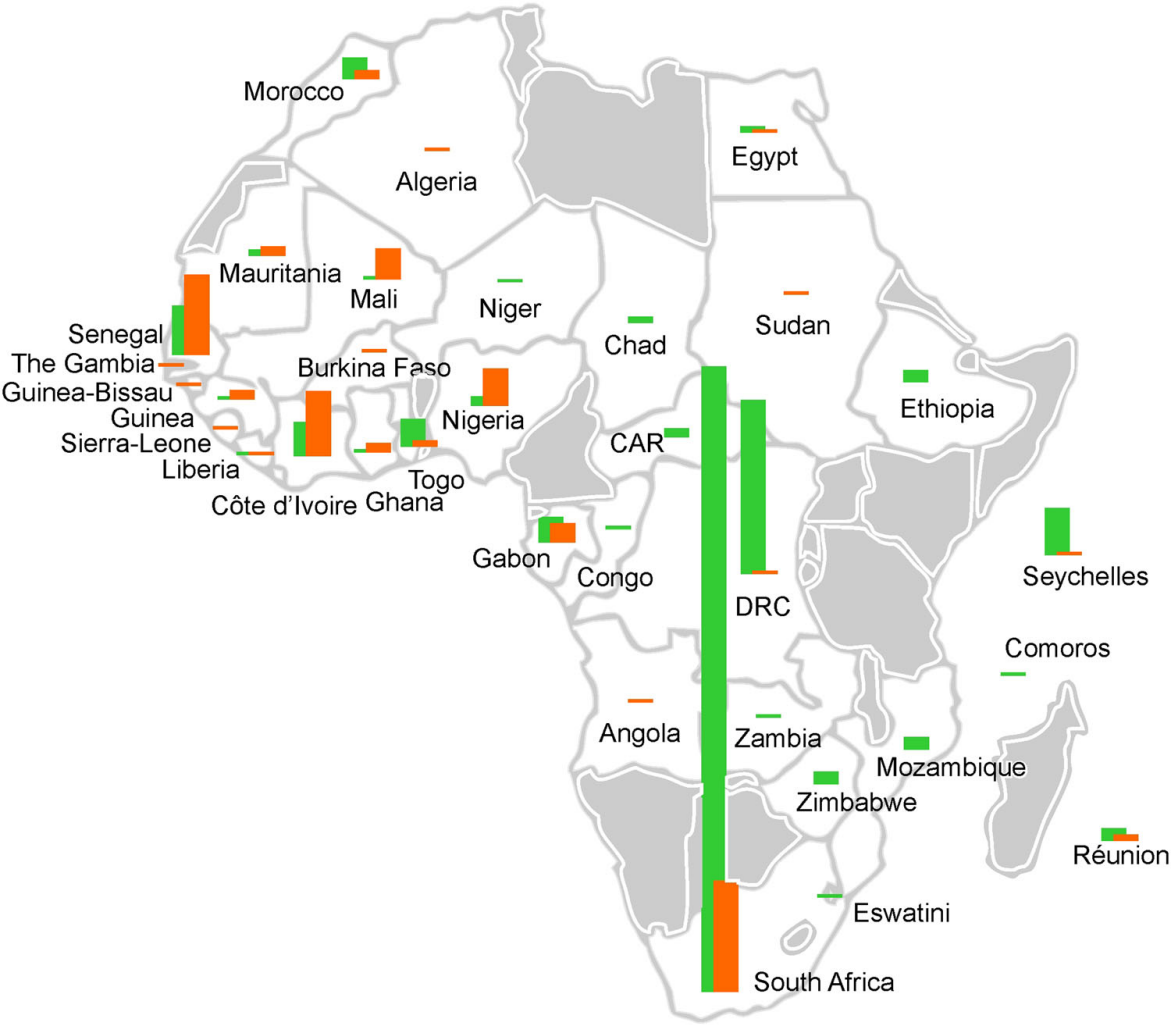
**ATL (orange) and TSP/HAM (green) cases as reported in 2023**.

HTLV‐1's pathogenicity manifests predominantly through ATLL and HAM/TSP, diseases mostly reported outside Africa due to diagnostic limitations and underreporting within the continent (Figure 2). ATLL is an aggressive hematological malignancy resulting from the malignant transformation of HTLV‐1‐infected CD4 + T‐cells, with latency periods often spanning decades. Prognosis remains grim, with median survival less than one year for acute forms, even in developed countries. Treatment options are limited and mainly palliative, although novel therapies targeting viral proteins and epigenetic regulators, as well as antibodies like Mogaluzimab, show promise in early clinical evaluation.^10^, ^11^, ^12^ However, recent research from Japan and Europe suggests that bone marrow transplantation with sibling, unrelated match donors, and more recently with haplo‐identical donors may increase overall survival at 5 years up to 50% in responding patients.^13^ HAM/TSP represents a progressive neuroinflammatory disease that severely impairs mobility. Both conditions are underdiagnosed in Africa due to limited diagnostic capacity, low clinical awareness, and a lack of serological and molecular confirmatory tools such as Western blot, PCR‐based clonality assays, or proviral load quantification. Additionally, infective dermatitis associated with HTLV‐1 (IDH),^14^ which is a chronic, recurrent skin condition in children, has emerged as a sentinel marker of early HTLV‐1 infection. IDH remains poorly recognized in Africa despite its epidemiological significance, largely due to clinical misclassification and restricted access to HTLV‐1 diagnostics.

## BLOOD SAFETY AND THE UNADDRESSED RISK OF HTLV‐1 TRANSMISSION

One of the most critical public health challenges is the risk of HTLV‐1 transmission via blood transfusion.^15^, ^16^, ^17^ A global perspective on blood donor screening practices reveals stark disparities between high‐income countries and resource‐limited settings. In the United States, Europe, and Japan, HTLV‐1 screening of all or at least first‐time blood donors has become routine in most countries, although cost‐effectiveness is debated due to low prevalence (10^−^^5^ to 10^−^^4^).^17^ Conversely, in many African countries with endemic HTLV‐1, screening is largely absent due to economic constraints. This lack of systematic screening is concerning, given that transmission risk from infected blood can reach 28%–63%, with organ transplantation risk even higher at 87%.^17^ Limited evidence on the efficacy of leukoreduction for preventing HTLV‐1 transmission, together with the lack of pathogen‐inactivation technologies, continues to hinder transfusion safety, although leukoreduction may reduce transmission risk to below 10%.^18^ An important unresolved question is the frequency and clinical impact of HTLV‐1 infection in chronically transfused populations, such as patients with sickle cell disease (SCD), the most common genetic disorder in Africa.

## REGIONAL POLICY RESPONSES AND WHO ENGAGEMENT

Despite the virus's wide geographical distribution and severe health impacts, including increased all‐cause mortality and socio‐economic burden, the WHO technical guidelines on HTLV‐1 remain in development. Recognized as a priority, these forthcoming guidelines will provide much‐needed frameworks for surveillance, testing, prevention, and care. The WHO's Global Health Sector Strategy on HIV, Viral Hepatitis, and Sexually Transmitted Infections (2022–2030) now includes HTLV‐1, signaling an important step toward integration. However, implementation in the African context requires tailored strategies to address biopsychosocial determinants, healthcare infrastructure gaps, and population‐specific transmission dynamics.

## RECENT RESEARCH ADVANCES INFORMING CLINICAL AND PUBLIC HEALTH PRACTICE

Innovative research continues to shed light on HTLV‐1 transmission and oncogenesis. For example, a longitudinal study of simian T‐cell leukemia virus type 1 (STLV‐1) in Japanese monkeys revealed occult infections, with long‐term proviral persistence without seroconversion, challenging conventional diagnostic paradigms and suggesting that similar hidden reservoirs may exist in humans.^19^ Similar study models could be implemented in African laboratories. At the genetic level, understanding the evolution of the mutational landscape in HTLV‐1 carriers offers promise for improved early risk stratification. Deep sequencing of recurrently mutated genes in high‐risk individuals with elevated proviral loads has already uncovered mutational landscapes that may predict progression to ATLL in the UK and Japan, enabling future targeted early interventions with monoclonal antibodies and anti‐retroviral therapies combining Interferon and AZT and other potential antivirals.^20^, ^21^

## POLICY GAPS AND REGIONAL ADVOCACY: A SUMMARY OF THE AFRICAN HTLV‐1 POLICY BRIEF

The virus, though known for decades, remains profoundly neglected, particularly in regions where it is likely endemic.

Given the absence of a cure or vaccine, policy responses must prioritize prevention. These include screening of at‐risk groups such as blood and organ donors, pregnant women, individuals with STIs including HIV, and sexual contacts and family members of those living with the virus. Diagnostic workflows rely on ELISA or chemiluminescence assays followed by confirmatory tests such as Western blot or PCR. While rapid diagnostic tests (RDTs) are emerging, they require further validation for use in African populations.

Prevention strategies are well‐defined. Condom use remains the most effective approach to prevent sexual transmission. For vertical transmission, exclusive formula feeding or shortened breastfeeding periods (less than 3 to 6 months) are recommended in endemic countries. A clinical trial on the use of integrase inhibitors to prevent vertical transmission is underway in Brazil. To reduce the risk of transfusion transmission, measures such as universal or selective screening and leukoreduction are recommended but not widely implemented in Africa due to cost constraints.

We also note important developments at the global level. The World Health Organization (WHO) has recently included HTLV‐1 in its *Global Health Sector Strategy on HIV, Viral Hepatitis, and Sexually Transmitted Infections (2022–2030)*. In parallel, the Pan American Health Organization (PAHO) has integrated HTLV‐1 into its *Elimination of Mother‐to‐Child Transmission (EMTCT) Plus* Initiative, which aims to eliminate vertical transmission of HIV, syphilis, hepatitis B, and Chagas disease, and now includes HTLV‐1 among its target pathogens. In addition, in the latest WHO (2022) and ICC classifications of hematological malignancies, HTLV‐1–related lymphoproliferations remain well‐characterized entities.

To address the gaps in Africa, we urge WHO‐AFRO to take a leadership role in promoting awareness, integrating HTLV‐1 into existing maternal‐child health and STI programs, providing updated information through regional communication platforms, training health workers, supporting prevalence studies, and facilitating the virus's inclusion among neglected tropical diseases and related funding mechanisms. Having international guidelines on HTLV‐1, a priority need identified in the WHO technical report on HTLV in 2019, will be of utmost importance for the region. HTLV‐1 awareness may benefit from improvements in SCD management, particularly through enhanced blood transfusion screening and targeted education programs.

These recommendations offer a practical roadmap to elevate HTLV‐1 as a public health priority and integrate it within broader disease control strategies already underway in the region.

## CONCLUSION

**Figure 3:**
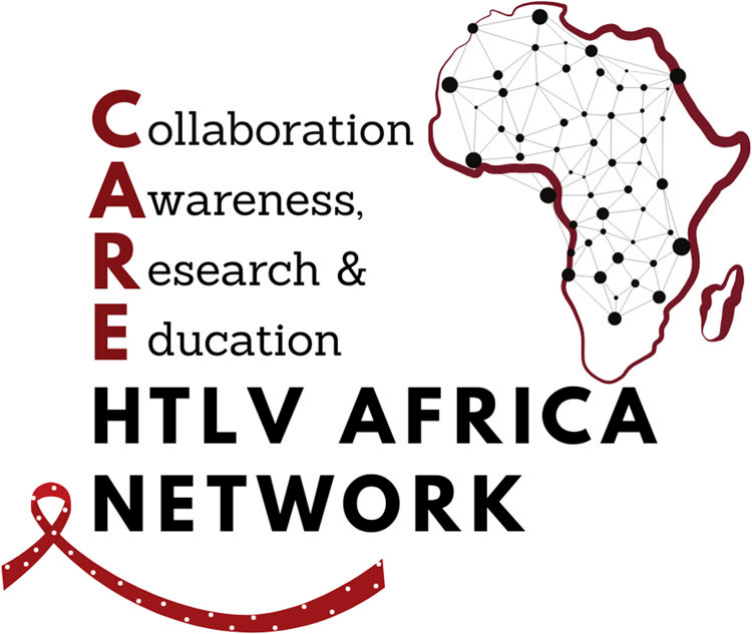
**A visual ID for the HTLV CARE Africa Network**.

At the conclusion of this workshop, we proposed the implementation of the HTLV CARE Africa Network for promoting Collaboration, Awareness, Research, and Education about HTLV in the region, and highlighted the pressing need for a coordinated public health response to HTLV‐1 in Africa (Figure 3). HTLV‐1 infection represents a silent but significant public health threat in Africa, one that demands urgent attention and action. Despite decades of scientific knowledge, the virus remains largely invisible in African healthcare and policy landscapes, with inadequate surveillance, limited diagnostic capacity, and virtually no routine blood donor screening in most endemic areas. This invisibility fuels continued transmission, delayed diagnosis, and poor outcomes from HTLV‐1‐associated diseases.

The actual geographical distribution of HTLV‐1 and the number of infected individuals remain largely unknown, especially in northern and eastern Africa. Large‐scale epidemiological surveys are critically needed to fill these gaps. Furthermore, local data on the clinical and epidemiological aspects of adult T‐cell leukemia/lymphoma (ATLL), HAM/TSP, infective dermatitis (ID), and other, yet unidentified, HTLV‐1–associated diseases, along with their impact on overall morbidity and mortality, are lacking across most African regions, hindering effective clinical and public health responses.

Africa's diverse transmission routes vary by region, but their relative contributions remain unclear. More comprehensive knowledge on transmission rates and routes is essential for targeted public health interventions. Central Africa exhibits remarkable HTLV‐1 genetic diversity, including multiple subtypes (b, d, e, f, g), with subtype b highly predominant, emphasizing the need for region‐specific research, diagnostic tools, and control strategies.

To meet these challenges, significant investments are needed in education, training, and information dissemination at all healthcare levels, supported by dedicated personnel and sustained funding. Capacity‐building efforts will empower local health systems to enhance diagnosis, prevention, and care, ultimately reducing HTLV‐1 transmission and disease burden.

Addressing these gaps requires a multipronged and integrated approach: expanded epidemiological studies to refine prevalence data, integration of HTLV‐1 testing into blood safety protocols, increased clinical training to improve recognition and management, and strong advocacy for policy development supported by WHO and regional health bodies. The lessons from global blood safety practices, coupled with emerging research on viral genetics and occult infections, provide an evidence base to inform these efforts.

Ultimately, reducing the burden of HTLV‐1 in Africa aligns with broader goals of strengthening health systems, improving transfusion safety, and tackling neglected infections that disproportionately affect vulnerable populations. It is time for HTLV‐1 to emerge from obscurity and enter the forefront of infectious disease control in Africa.

## AUTHOR CONTRIBUTIONS

**Jean‐Claude Twizere**: Conceptualization; funding acquisition; writing—original draft; supervision; writing—review and editing. **Carolina Rosadas**: Funding acquisition; writing—review and editing. **Maureen Kidiga**: Writing—review and editing. **Fatumata Djalo**: Writing—review and editing. **Edward L. Murphy**: Writing—original draft; writing—review and editing. **Augustin Mouinga‐Ondeme**: Writing—original draft; writing—review and editing. **Marion Vermeulen**: Writing—review and editing. **Louine Morrell**: Writing—review and editing. **Saliou Diop**: Writing—review and editing. **Sibusiso Maseko**: Writing—review and editing. **Peregrine Sebulime**: Writing—review and editing. **Andrews Akwasi Agbleke**: Writing—review and editing. **Boineelo Fundisi**: Writing—review and editing. **Espérance Umumararungu**: Writing—review and editing. **Patricia Watber**: Writing—review and editing. **Carol Hlela**: Writing—review and editing. **Egídio Nhavene**: Writing—review and editing. **Thato Chidarikire**: Writing—review and editing. **Olivier Hermine**: Writing—review and editing; funding acquisition; writing—original draft; supervision. **Antoine Gessain**: Funding acquisition; writing—original draft; writing—review and editing; supervision.

## CONFLICT OF INTEREST STATEMENT

The authors declare no conflicts of interest.

## FUNDING

This study was supported by International Retrovirology Association, Fonds De La Recherche Scientifique ‐ FNRS (40033041), Medical Research Council (Grant number MR/X022358/1), the Inidex Grex Laboratory of Excellence on Red Cells, Imperial College London (UK), and NIHR Imperial Biomedical Research Centre.

## Data Availability

Data sharing is not applicable to this article as no datasets were generated or analyzed during the current study.
